# Imaging of Cellular Oxidoreductase Activity Suggests Mixotrophic Metabolisms in *Thiomargarita* spp.

**DOI:** 10.1128/mBio.01263-17

**Published:** 2017-11-07

**Authors:** Jake V. Bailey, Beverly E. Flood, Elizabeth Ricci, Nathalie Delherbe

**Affiliations:** Department of Earth Sciences, University of Minnesota, Minneapolis, Minnesota, USA; University of California, Berkeley

**Keywords:** *Beggiatoa*, *Thiomargarita*, chemolithotrophy

## Abstract

The largest known bacteria, *Thiomargarita* spp., have yet to be isolated in pure culture, but their large size allows for individual cells to be monitored in time course experiments or to be individually sorted for omics-based investigations. Here we investigated the metabolism of individual cells of *Thiomargarita* spp. by using a novel application of a tetrazolium-based dye that measures oxidoreductase activity. When coupled with microscopy, staining of the cells with a tetrazolium-formazan dye allows metabolic responses in *Thiomargarita* spp. to be to be tracked in the absence of observable cell division. Additionally, the metabolic activity of *Thiomargarita* sp. cells can be differentiated from the metabolism of other microbes in specimens that contain adherent bacteria. The results of our redox dye-based assay suggest that *Thiomargarita* is the most metabolically versatile under anoxic conditions, where it appears to express cellular oxidoreductase activity in response to the electron donors succinate, acetate, citrate, formate, thiosulfate, H_2_, and H_2_S. Under hypoxic conditions, formazan staining results suggest the metabolism of succinate and likely acetate, citrate, and H_2_S. Cells incubated under oxic conditions showed the weakest formazan staining response, and then only to H_2_S, citrate, and perhaps succinate. These results provide experimental validation of recent genomic studies of *Candidatus* Thiomargarita nelsonii that suggest metabolic plasticity and mixotrophic metabolism. The cellular oxidoreductase response of bacteria attached to the exterior of *Thiomargarita* also supports the possibility of trophic interactions between these largest of known bacteria and attached epibionts.

## IMPORTANCE

The metabolic potential of many microorganisms that cannot be grown in the laboratory is known only from genomic data. Genomes of *Thiomargarita* spp. suggest that these largest of known bacteria are mixotrophs, combining lithotrophic metabolism with organic carbon degradation. Our use of a redox-sensitive tetrazolium dye to query the metabolism of these bacteria provides an independent line of evidence that corroborates the apparent metabolic plasticity of *Thiomargarita* observed in recently produced genomes. Finding new cultivation-independent means of testing genomic results is critical to testing genome-derived hypotheses on the metabolic potentials of uncultivated microorganisms.

## INTRODUCTION

Sulfide-oxidizing bacteria of the family *Beggiatoaceae* contain some of the largest known bacteria (1–5), with individual cells of the genus *Thiomargarita* spp. reaching millimetric diameters (3, 5, 6). Dense communities of these organisms on the seafloor make up some of the most spatially extensive microbial mat ecosystems on earth (4, 7). The large sulfur bacteria are primarily chemolithotrophs or mixotrophs that live at interfaces between nitrate or oxygen and hydrogen sulfide (8–12). These bacteria can have a substantial influence on the biogeochemical cycling of sulfur, nitrogen, phosphorus, and carbon in diverse environmental settings (11, 13–15). Despite their large size and biogeochemical significance, the physiologies and ecologies of these bacteria remain incompletely understood, in part because the large sulfur bacteria are not presently isolated in pure culture. At present, *Thiomargarita* spp. can only be maintained long-term in their natural sediments, not in axenic isolation or even as mixed communities in a defined microbial medium. Therefore, approaches to studying *Thiomargarita*’s physiology have focused on culture-independent methods. Schulz and de Beer used microsensors to measure O_2_ and sulfide gradients influenced by *Thiomargarita* metabolism (16), and more recently, (meta)genomic and metatranscriptomic approaches have been used to investigate their genetic potential (17, 18) and response to environmental perturbations (19). Here we add to these findings by using a novel approach to the study of large sulfur bacteria that employs tetrazolium redox dyes.

Tetrazolium dyes are widely used to measure the metabolic activity of cells, including both bacteria (20–22) and eukaryotes (23), growing on a variety of metabolic substrates. In their oxidized form, soluble tetrazolium salts generally result in a colorless or weakly colored solution. Cellular oxidoreductases, such as NADH dehydrogenase, reduce tetrazolium salts to an intensely colored formazan product that can be observed qualitatively or measured quantitatively in a variety of colorimetric metabolic assays (24–26). These dyes can be used to measure catabolic metabolic activity even in the absence of observable cell division. Typically, tetrazolium dyes are applied to bacteria in culture and measured via the color response of the bulk medium and culture in a microplate (27). We sought to apply a tetrazolium dye approach to investigate metabolism in *Thiomargarita* spp. However, *Thiomargarita* spp. are not in pure culture, and these large cells are covered with communities of smaller attached bacteria (12). Our initial attempts to analyze our custom tetrazolium microplate assays via spectrophotometry failed to differentiate between the metabolism of *Thiomargarita* and that of the bacteria attached to it. We then employed a microscopy-based approach to image the tetrazolium color change associated with individual *Thiomargarita* cells so as to differentiate metabolic responses of *Thiomargarita* cells from those of attached epibiont cells. Here we report on the cellular oxidoreductase response of *Thiomargarita* cells to several organic and inorganic substrates under oxic, hypoxic, and anoxic conditions.

## RESULTS AND DISCUSSION

### Tetrazolium dye staining and the metabolism of lithotrophic substrates.

**FIG 1:**
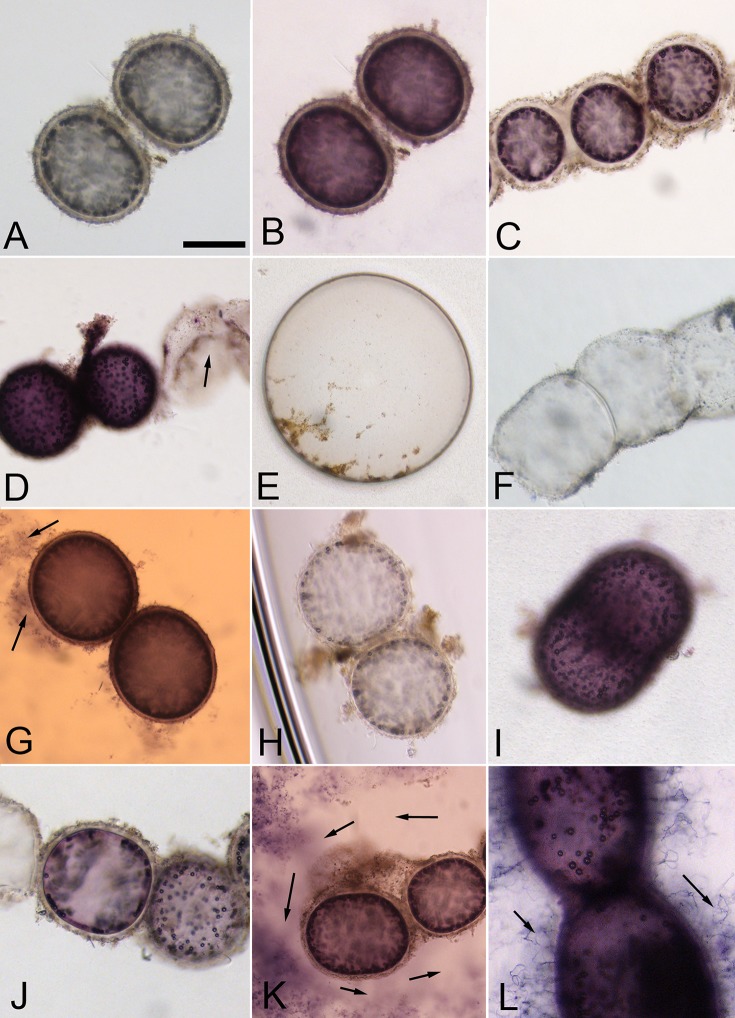
Cellular oxidoreductase activity reduces colorless tetrazolium to a purple formazan product. (A) Initially, *Thiomargarita* sp. cells incubated in the redox dye are colorless and the medium is light green. (B) Many intact cells under specific treatment conditions, presumably those that are metabolically active, stain a deep purple, and the extracellular medium changes to colorless or light pink, typically within 2 days. Panel B shows cells incubated under anoxic conditions in the presence of succinate. Staining of metabolically active cells was spatially separate and distinct from that of the surrounding sheath material (C). Collapsed or damaged cells at the time of incubation did not exhibit a color change (D), showing exposure to acetate and hydrogen, respectively, under anoxic conditions. Staining of metabolically active cells was distinct in intensity from that of control diatom frustules (E) and control sheath material (F). Upon exposure to H_2_S, the extracellular medium assumed an orange hue that was readily distinguishable from the purple color change in the *Thiomargarita* sp. cells (G) and in biofilms of attached epibiont bacteria (arrows). Under anoxic conditions, cells and medium showed the most extensive response, with very little color change observed with no additional electron donor added (H) and a strong staining response with the addition of other substrates such as H_2_ under anoxic conditions (I and J). In some cases, staining of extracellular bacteria was present as a diffuse stained cloud composed of small cells within the well. A zone characterized by absence of staining and cells in the immediate vicinity of the *Thiomargarita* cells suggests some sort of inhibition of these small bacteria (arrows in panel K). In other cases, such as exposure to thiosulfate under anoxic conditions, as shown here, stained filamentous epibionts could be observed anchored to the *Thiomargarita* cell/sheath (L). All images were taken at ×400 magnification. Scale bar in panel A, ~100 μm.

**FIG 2:**
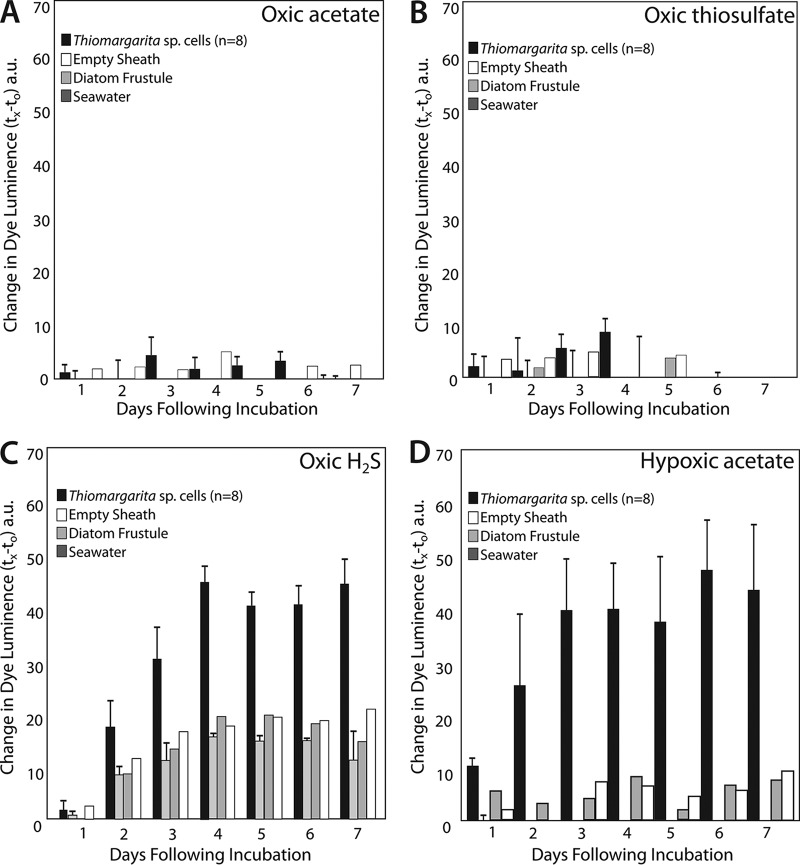
*Thiomargarita* cells exhibited very little staining response to acetate (A), thiosulfate (B), and other substrates under oxygenated conditions relative to that of controls. However, H_2_S (C) and citrate (not shown) did induce a statistically significant staining response under oxic conditions. Potentially significant staining was observed under hypoxic conditions in the presence of acetate (D), succinate, citrate, and H_2_S. Plotted here is the mean change in reciprocal intensity of luminance relative to that at day zero. Error bars indicate the standard error of the mean of the *Thiomargarita* incubations or controls. a.u., arbitrary units.

**FIG 3:**
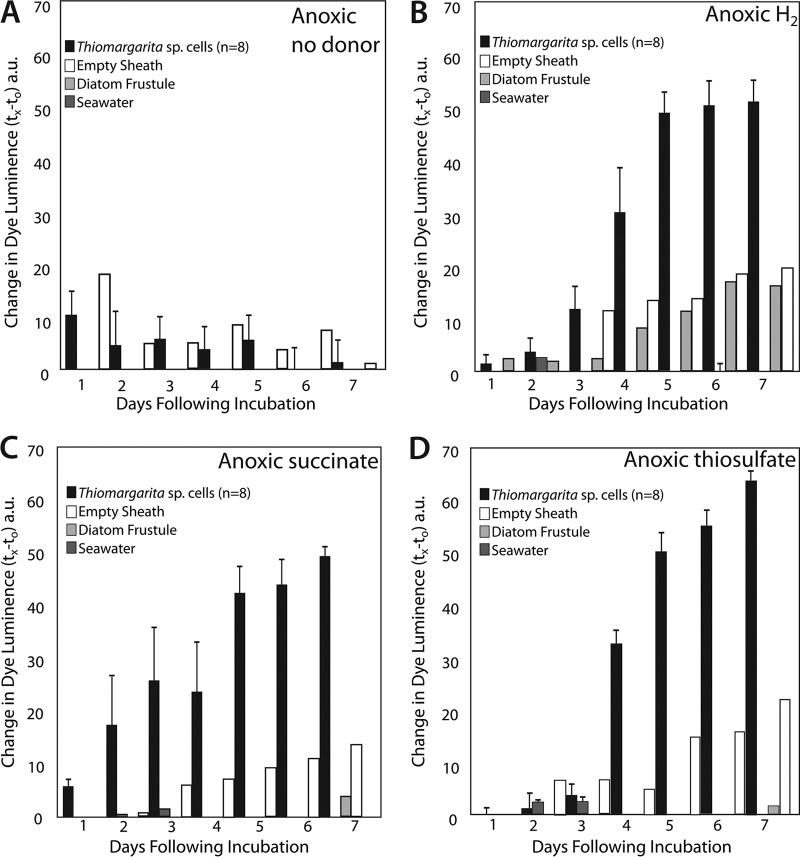
*Thiomargarita* cells and controls showed a weak response under anoxic conditions with the addition of a substrate that could not be differentiated from controls (A). With the addition of H_2_ (B), succinate (C), thiosulfate (D), H_2_S, formate, acetate, citrate, and formate (not shown), a statistically significant staining response was observed under anoxic conditions. Plotted here is the mean change in the reciprocal intensity of luminance relative to that at day zero. Error bars indicate the standard error of the mean of the *Thiomargarita* incubations or controls. a.u., arbitrary units.

*Thiomargarita* sp. cells exhibited apparent metabolic responses to a variety of substrates, as indicated by formazan staining that was localized within the cell (Fig. 1) and that increased in intensity over the duration of the 7-day experiment (Fig. 1A and B, 2, and 3). Maximum staining generally occurred within 4 to 6 days (Fig. 2 and 3).

Intense color changes were restricted to the *Thiomargarita* cell itself and did not extend to the sheath (Fig. 1B, C, and J), except in cases where epibiont bacteria were stained as described below or in the case of sulfide treatments, also described below. *Thiomargarita* cells sometimes underwent spontaneous collapse, as is observed to occur in unamended freshly collected natural *Thiomargarita* populations. Collapsed or otherwise damaged cells at the time of incubation never showed a color change (Fig. 1D), though some cells that did stain early in the experiment were sometimes observed to collapse in the later stages of the experiment. These observations suggest that formazan staining is a good indicator of initial cell viability, but that oxidoreductase activity alone was not sufficient to prevent eventual cell collapse in some specimens. Staining of controls, which included cell rinse water (not shown), diatom frustule (Fig. 1E), and empty sheaths (Fig. 1F), exhibited very low intensities compared with *Thiomargarita* cell staining (Table 1; Fig. 2 and 3).

**TABLE 1:** Mean positive change in the reciprocal intensity of luminance on day 7 relative to that at time zero^a^

Conditions and substrate	Mean positive change in reciprocal luminance intensity ± SEM (*n*)			
	*Thiomargarita* cells	Sheath control	Diatom control	Water controls
Oxic				
No donor added	3.503 ± 3.0 (8)	0	0	0 ± 0.2 (2)
Succinate	3.4 ± 2.1 (8)	5.8	0.7	0 ± 3.2 (2)
Thiosulfate	All cells collapsed (0)	0	0	0 ± 4.7 (2)
Acetate	0 ± 1.9 (8)	2.7	0	0 ± 1.8 (2)
Citrate	**13.0 ± 2.1 (8)**	4.0	2.9	0 ± 2.4 (2)
Formate	All cells collapsed (0)	0	5.7	0 ± 0.2 (2)
H_2_S	**45.3 ± 4.6 (8)**	21.2	15.1	11.3 ± 3.8 (2)
Hypoxic				
No donor added	5.1 ± 2.3 (8)	0	0.8	3.0 ± 0.6 (2)
Succinate	**24.7 ± 2.1 (8)**	0	3.1	0 ± 0.4 (2)
Thiosulfate	All cells collapsed (0)	5.2	0.7	0 ± 0.3 (2)
Acetate	44.1 ± 12.1 (8)	9.4	7.7	0 ± 0.6 (2)
Citrate	30.6 ± 4.3 (4)	1.6	0	0 ± 0.4 (2)
Formate	4.0 ± 1.6 (8)	3.4	4.7	0 ± 0. (2)
H_2_S	61.5 ± 4.5 (6)	39.4	51.0	38.4 ± 8.0 (2)
Anoxic				
No donor added	1.104 ± 4.6 (8)	2.0	0	0 ± 0.6 (2)
Succinate	**50.7 ± 1.3 (3)**	13.5	3.9	0 ± 5.6 (2)
Thiosulfate	**73.4 ± 2.0 (8)**	25.8	1.9	0 ± 0.7 (2)
Acetate	**42.2 ± 3.9 (8)**	12.3	4.7	0.1 ± 1.1 (2)
Citrate	**37.5 ± 4.0 (8)**	22.3	0	3.0 ± 0.3 (2)
Formate	**42.2 ± 6.2 (8)**	2.6	13.5	0.4 ± 0.1 (2)
H_2_S	**40.9 ± 5.6 (8)**	25.1	20.3	11.2 ± 2.8 (2)
H_2_	**62.0 ± 5.1 (8)**	23.6	19.6	0 ± 0.5 (2)

^a^ Values that are highly statistically different (*P* < 0.005, Student *t* test) from the mean of the four control samples are in boldface type. Values that are marginally statistically different (*P* < 0.05, Student *t* test) from the mean of the four control samples are underlined. Negative values were used to calculate standard errors but are reported as 0 to indicate no positive change.

Sulfur bacteria such as *Thiomargarita* are known for their ability to oxidize H_2_S by using O_2_ or nitrate as an electron donor (3, 12). The intense tetrazolium dye staining of cells exposed to H_2_S under both oxic and anoxic conditions was highly statistically supported as being distinct from control staining (*P* < 0.005, Student *t* test), while staining under sulfidic hypoxic conditions was marginally statistically distinct from control staining (*P* < 0.05, Student *t* test) (Table 1). The genomes of *Thiomargarita* spp. contain genes for the oxidation of H_2_S via a sulfide:quinone oxidoreductase and/or flavocytochrome *c* (17). In the treatments containing H_2_S, an orange color change was noted in the medium (Fig. 1G). The orange color change, which occurred only in the presence of H_2_S, was distinguishable from the typical formazan purple color change that occurs within the cells (Fig. 1G). This orange coloration is likely the result of the abiotic reduction of tetrazolium by H_2_S. Despite the sulfide treatments having a higher background than other treatments, a dark purple color change in the cell could be differentiated from the orange background (Fig. 1G and 2C; Table 1).

Many sulfur-oxidizing bacteria are also known to be able to oxidize other sulfur-containing substrates such as thiosulfate. Thiosulfate addition to the experimental wells resulted in an increasingly strong formazan staining response under anoxic conditions (*P* < 0.005, Student *t* test) (Fig. 3D). However, significant staining was not observed with thiosulfate addition under oxic and hypoxic conditions (Table 1). Instead, cells were observed to collapse (Fig. 2B). All currently available genomes of *Beggiatoaceae* contain the genes for thiosulfate oxidation via a partial *sox* system (*soxABXY*) for the oxidation of thiosulfate to intracellularly stored sulfur granules (17, 18). Thiosulfate is not as strong a reductant as H_2_S, which may have left *Thiomargarita* cells susceptible to oxidative stress under oxic and hypoxic conditions. Indeed, cell collapse was common with a variety of metabolic substrates under oxic conditions. Oxic treatments with H_2_S were the only exception.

In addition to reduced sulfur compounds, some sulfur-oxidizing bacteria are also known to use H_2_ as an electron donor for lithotrophic metabolism (28). Our staining results also showed strong statistical support (*P* < 0.005, Student *t* test) for formazan staining relative to controls when the cells were incubated in an anaerobic glove box containing 3% H_2_ (Fig. 1D, I, and J and 3B). Cells incubated without other supplied electron donors in an anoxic chamber without H_2_ showed no color change (Fig. 1H and 3A). Recently, a chemolithotrophic strain of *Beggiatoa* sp., 35Flor, was found to use H_2_ as an electron donor under oxygenated conditions (29). 35Flor was not observed to oxidize H_2_ under anaerobic conditions, but it does not store nitrate to serve as an electron acceptor as *Thiomargarita* does. In addition to the microsensor studies that show H_2_ consumption by *Beggiatoa* sp. strain 35Flor, the genomes of *Candidatus* Thiomargarita nelsonii Thio36 and Bud S10 both contain genes for Ni-Fe hydrogenase (17, 18), as do those of other sulfur-oxidizing bacteria (30, 31). The source of H_2_ for *Thiomargarita* in nature is not clear, but fermentation by epibionts or other bacteria in the environment is a possible source.

### Organic acid metabolism.

In addition to their canonical lithotrophic metabolism, some representatives of the family *Beggiatoaceae* are known to metabolize organic acids (32). Organic acid degradation may be a common trait among sulfur-oxidizing gammaproteobacteria, since the generation of NADPH through the tricarboxylic acid (TCA) cycle would coincide with the assimilation of organic acids and would thus reduce the energetic costs of carbon fixation (33, 34). The genomes of *Ca.* Thiomargarita nelsonii Bud S10 and Thio36 and other members of the family *Beggiatoaceae* contain genes for a complete TCA cycle, including the gene for NADH dehydrogenase I (17, 18, 35, 36), and while succinate dehydrogenase is not electrochemically favored under most anaerobic conditions, it is tightly coupled with respiratory nitrate and nitrite reduction (37). Nitrate was provided in the medium (100 µM), and nitrate is also stored in the internal nitrate vacuole.

All of the organic acids tested here yielded positive staining results under anoxic conditions, but staining results with organic acid exposure were more variable under hypoxic and aerobic conditions. We observed a positive staining response with the addition of citrate under oxic, hypoxic, and anoxic conditions and succinate under hypoxic and anoxic conditions (Table 1). The utilization of exogenous organic acids requires specific inner membrane transporters. The genomes of a few members of the family *Beggiatoaceae* encode putative citrate transporters (Ga0060138_111857, BGP_2612), and a partial putative citrate transporter is present in *Ca.* Thiomargarita nelsonii Thio36 (Thi036DRAFT_00068870). Succinate is usually transported by proteins encoded by either one of two three-gene clusters, *dctABD*, where *dctA* is a permease and *dctBD* is a two-component system for the activation of transcription (38), or *kgtPSR* (39), or via a tripartite ATP-independent periplasmic (TRAP) transporter, *dctPQM* (40). The two available *Thiomargarita* genomes contain a *dctB* gene but lack *dctAD*, although *dctB* is located near the terminal end of a contig in the genome of Bud S10. While freshwater strains of *Beggiatoa* (*Beggiatoa leptomitoformis* D-402 and *B. alba* B18LD) possess putative *dctPQM* genes, annotated genes for C_4_-dicarboxylate transporters are lacking in all marine strains. Thus, the capacity to transport succinate via known transporters appears to be lacking in marine members of the family *Beggiatoaceae*. However, the marine strains do possess a number of uncharacterized TRAP transporters that might serve as future targets for the characterization of C_4_-dicarboxylate metabolism.

Acetate is another organic acid that is thought to be metabolized by certain sulfur bacteria. Acetate can be metabolized by the TCA and glyoxylate cycles, as well as putative alternate pathways that make carboxylic acids as intermediates for biosynthesis, but that may not yield energy to the cell (41). The microelectrode respiration experiments of Schulz and de Beer showed the stabilization of oxygen-sulfide gradients around *Thiomargarita* when acetate was added to the experiments, although acetate itself was not found to steepen the oxygen gradient (16). Our formazan dye staining results show no statistical support for acetate use under oxic conditions, which is consistent with the results of Schulz and de Beer. However, our results also show marginal statistical support for acetate metabolism under hypoxic conditions and strong support for acetate use under anoxic conditions (Table 1; Fig. 2D). Acetate metabolism is preceded by the activation of acetate by acetate kinase and phosphotransacetylase (42) (known as the *ack-pta* pathway) and/or via acetyl coenzyme A synthetase (known as the ACS pathway). The *ack-pta* pathway occurs in the freshwater strains of members of the family *Beggiatoaceae* but not in the marine strains. However, almost all members of the family *Beggiatoaceae* possess the ACS pathway (18) and an acetate/cation symporter (e.g., Ga0063879_05139) (36). These genomic features are consistent with the ability of *Thiomargarita* spp. to take up and metabolize acetate from the environment, but our staining results suggest that this may occur only under anoxic and perhaps hypoxic conditions in *Thiomargarita* spp. The activity of cellular oxidoreductases that reduce tetrazolium to formazan under acetate exposure, as we observed here under hypoxic and anoxic conditions, suggests that acetate is metabolized by a pathway such as the TCA cycle that contributes to energy production, as well as biosynthetic intermediates. In future studies, isotopically labeled organic substrates and their assimilation into *Thiomargarita* could be used to determine the relative contributions of those substrates to biosynthesis versus energy production.

Formate is both a waste product of fermentation and a potential electron donor that can be used for both aerobic and anaerobic respiration (43). The genomes of *Ca.* Thiomargarita nelsonii Thio36 and *Beggiatoa* strain PS both contain genes that code for subunits of formate dehydrogenase. In addition to anaerobic respiration, the energy-yielding reaction under anaerobic conditions is the formate-assisted cleavage of pyruvate via a pyruvate formate lyase enzyme. The genomes of *Ca.* Thiomargarita nelsonii Thio36 and Bud S10 both contain three annotated pyruvate formate lyase genes, and these genes occur in most other representatives of the family *Beggiatoaceae*. The transport of exogenous formate could be mediated by a formate/nitrate transporter (FocA), which is present in most marine strains of *Beggiatoaceae*, including both *Ca.* Thiomargarita nelsonii genomes.

Although *Thiomargarita* spp. are thought to be more oxygen tolerant than other marine vacuolate sulfur bacteria, such as *Ca.* Maribeggiatoa and *Ca.* Marithioploca (16), the results presented here suggest that while *Thiomargarita* spp. may tolerate oxygen exposure, their metabolism(s) is most versatile under anoxic conditions. This is perhaps unsurprising, given that *Thiomargarita* bacteria on the Namibian shelf are found primarily in sediments that are anoxic for much of the year (44). During the collection of the samples used in our experiments, no oxygen was detectable in the lower water column, as measured by Winkler titration, and in core-top waters, as measured with a handheld oxygen meter. Limited tolerance to O_2_ is also suggested by recent genomic results for most members of the family *Beggiatoaceae*. All *Beggiatoaceae* strains have *cbb*_3_-type cytochrome *c* oxidase, and some also possess *bd*-type cytochromes, both of which are specific to hypoxic conditions. However, both *Ca.* Thiomargarita nelsonii Thio36 and *Beggiatoa* strain PS possess the more oxygen-tolerant cytochrome *c* oxidase (*coxABC*, *cyoE*), which suggests greater O_2_ tolerance in some strains of *Beggiatoaceae*. Catalase, which is used to ameliorate oxidative stress, is not present in the genomes of marine *Beggiatoaceae* and occurs only in the freshwater strains. Most *Beggiatoaceae* genomes contain a superoxide dismutase and a cytochrome *c* peroxidase, while most marine strains also possess a desulfoferredoxin. The enzymes coded for by these genes may provide protection against O_2_ and reactive oxygen species during periodic exposure to O_2_. Additionally, H_2_S, which can scavenge reactive oxygen species (45), may serve as an extracellular antioxidant under conditions in which sulfide is fluxing into oxygenated waters. Under anoxic conditions, oxidized forms of inorganic nitrogen, whether exogenous or stored within the vacuole, serve as terminal electron acceptors (3, 46). *Ca.* Thiomargarita nelsonii Bud S10 (17) and Thio36 (18) both possess a complete denitrification pathway that includes both membrane-bound (*nar*) and periplasmic (*nap*) nitrate oxidoreductases and the capacity to reduce nitrite to ammonium (*nirBD*). Thus, our staining results are consistent with the genomes and the canonical knowledge of nitrate being used as an electron acceptor by certain members of the family *Beggiatoaceae*.

### Epibiont microbial cells.

In our experiments, we undertook imaging of individual formazan-stained *Thiomargarita* cells in order to differentiate *Thiomargarita* metabolism from that of attached bacteria. These attached bacteria exhibited staining spatially distinct from that of *Thiomargarita* under the microscope that could not be differentiated with a typical microplate assay. Our microscope-based imaging approach had the added benefit of allowing us to observe the discrete staining of epibiont biofilms and filaments associated with *Thiomargarita* (Fig. 1G and L). In some cases, stained filamentous epibionts could be observed anchored to the *Thiomargarita* cell/sheath (e.g., Fig. 1L, arrows). In particular, dense accumulations of filamentous bacteria (Fig. 1L) exhibited intense staining when exposed to H_2_ under anoxic conditions. While filamentous bacteria were observed in some control wells and in zones of the well distal to *Thiomargarita*, their accumulation was far denser in the vicinity of *Thiomargarita* cells (Fig. 1L).

We also observed under anoxic conditions in the presence of H_2_S that small bacteria that stained purple were present throughout the medium, except in clear cell-free zones we observed immediately surrounding *Thiomargarita* cells and chains (Fig. 1K, arrows). Additional studies are needed to identify these epibiont bacteria and determine the nature of their trophic interactions (if any) with *Thiomargarita* spp.

### Conclusions.

Our observations of oxidoreductase-mediated formazan staining in *Thiomargarita* cells exposed to a variety of organic and inorganic substrates are consistent with recent genomic results that suggest metabolic plasticity in *Ca.* Thiomargarita nelsonii. There are, however, some limitations and caveats in drawing broad conclusions from our results. It is possible that the incubation medium formulations or concentrations we used here, or other aspects of the experimental design, resulted in responses from *Thiomargarita* bacteria that do not represent their metabolic activities in nature. Yet the results presented here are broadly consistent with genomic data currently available for *Ca.* Thiomargarita nelsonii and consistent with the sediment-hosted habitat of these bacteria that is anoxic for much of the year. We used both the spherical *Thiomargarita* cells that are typical of *Thiomargarita namibiensis* and the cylindrical forms that are more typical of *Ca.* Thiomargarita nelsonii for this study (1). However, the large number of cells used in the experiment, and the difficulty in amplifying the *Thiomargarita* 16S rRNA gene because of the multiple introns contained therein, prevented a detailed phylogenetic characterization of the cells used for the assay (47). Therefore, we cannot say that our results apply broadly to the multiple candidate *Thiomargarita* species that can sometimes co-occur in sediments off Namibia (1, 48). While cultivation will ultimately be necessary for rigorous testing of the physiologies of *Thiomargarita* spp., for now, these results provide additional evidence in support of genomic and microsensor findings of metabolic versatility and/or mixotrophy in the genus *Thiomargarita* and suggest cultivation approaches that include both reduced sulfur compounds and organic substrates perhaps under extremely low oxygen conditions.

Tetrazolium/formazan redox dyes have long been used to study bacteria, primarily in plate-based assays. Our expansion of the use of these dyes to the microscopic examination of individual stained cells may be broadly useful for assaying metabolism in mixed communities and other uncultivated microorganisms and for validation of genome-derived assessments of physiological capabilities.

## MATERIALS AND METHODS

### Sample collection.

*Thiomargarita* sp. cells were collected from organic-rich sediments on the Atlantic shelf near Walvis Bay, Namibia (23°00.009′N 14°04.117′E) with a multicorer on board the R/V *Mirabilis*. All of the cells used in the experiments described here were collected at a depth of 1 to 3 cm beneath the sediment-water interface. *Thiomargarita* sp. cells were stored in their host sediments with overlying core top water in closed 50-ml centrifuge tubes at 4°C and protected from direct light for 3 weeks.

### Incubation experiments with tetrazolium staining.

Chains and clusters of *Thiomargarita* sp. cells were rinsed three times in 0.2-µm-filtered artificial seawater before being added to an incubation medium in 96-well microplates. The purpose of these rinsing steps was to remove loosely attached smaller bacteria from the exterior of *Thiomargarita* cells and sheaths. These washing steps did not remove tightly attached or sheath-embedded epibionts, as confirmed by microscopy and described above. One *Thiomargarita* cell, cell cluster, or cell chain was added to each individual well of the 96-well plate. Each substrate treatment contained eight wells with *Thiomargarita* cells, as well as four control wells without *Thiomargarita* cells. Two of these control wells contained 20 μl of the first saltwater bath used to rinse the *Thiomargarita* cells, added to 180 μl of medium, as a control that contains cells found loosely attached to *Thiomargarita* cells. Wells containing empty diatom frustules picked by pipette from the same samples as the *Thiomargarita* cells were also used as negative controls to monitor the color change of xenic biological surfaces from the same environment. A third control type consisted of empty mucus sheaths that were produced by *Thiomargarita* cells but no longer contained the cells. These empty sheaths are common in *Thiomargarita*-rich sediments and can be readily identified by the shape of the chains that is preserved as void space in the sheath material.

The incubation medium was designed to maintain metabolizing *Thiomargarita* cells and to provide basic elemental constituents and nutrients based on similar base medium recipes for phylogenetically related taxa (34). The incubation medium included the following constituents (liter^−1^): 34 g of NaCl, 0.112 g of CaCl_2_, 0.008 g of NH_4_NO_3_, 0.5 g of KCl, 1.46 g of MgSO_4_, 20 ml of 1 M MOPS [3-(*N*-morpholino)propanesulfonic acid] buffer (pH adjusted to 7.8, final concentration, 20 mM), 1 ml of 1,000× potassium phosphate buffer (pH 7.6), 1 ml of 1,000× vitamin B_12_, 1 ml of a 1,000× vitamin solution, and 10 ml of a 100× trace element solution. The vitamin solution contained (liter^−1^) 10 mg of riboflavin and 100 mg each of thiamine HCl, thiamine pyrophosphate, l-ascorbic acid, d-Ca-pantothenate, folic acid, biotin, lipoic acid, nicotinic acid, 4-aminobenzoic acid, pyridoxine HCl, thiotic acid, NAD, and inositol dissolved in 100 ml of 10 mM KPO_4_ buffer (pH 7). The trace metal solution contained (liter^−1^) 0.1 g of FeCl_2_ ⋅ 2H_2_O, 0.03 g of H_3_BO_3_, 0.1 g of MnCl_2_, 0.1 g of CoCl_2_ ⋅ 6H_2_O, 1.5 g of nitrilotriacetic acid, 0.002 g of CuCl_2_ ⋅ 6H_2_O, 0.024 g of NiCl_2_ ⋅ 6H_2_O, 0.144 g of ZnSO_4_ ⋅ 7H_2_O, 0.036 g of NaMoO_4_, 0.025 g of Na-vanadate, 0.010 g of NaSeO_3_, and 0.01 g of NaWO_4_ ⋅ 2H_2_O. NaHCO_3_ was added to each base medium to a final concentration of 3 mM for the oxic and anoxic stocks or 40 mM for the hypoxic treatments. The medium was sterilized by filtration through 0.22-μm membrane, and the final pH was 7.9 to 8.0.

Potential electron donors acted as experimental variables and included H_2_, H_2_S, thiosulfate, succinate, acetate, citrate, and formate. All electron donors except H_2_ and H_2_S were supplied at a final concentration of 1 mM. H_2_ was supplied by shaking the plates in a Coy anaerobic chamber containing a 3% H_2_, 97% N_2_ atmosphere. H_2_S was supplied by the daily addition of 10 μl of the freshly neutralized 4 mM sodium sulfide delivered by syringe. The tetrazolium redox dye mixture we used, which is known by the commercial name Dye H (Biolog catalog no. 74228), was added to the medium at a final 1× working strength just prior to cell incubation.

Microplates were placed on orbital shakers at 50 rpm. One microplate was maintained under benchtop atmospheric conditions, one microplate was placed in a Coy hypoxic chamber with 5% atmospheric level O_2_ and ~5% total CO_2_, one microplate was placed in an H_2_-free anaerobic chamber (NextGen; Vacuum Atmospheres Company, Hawthorne, CA), and one microplate was placed in a Coy anaerobic chamber containing a mixture of 97% N_2_ and 3% H_2_. All plates were maintained in plastic containers with an open aperture to allow free exchange of gases for the week-long duration of the experiment. The plastic chambers that housed the microplates contained moistened paper towels to inhibit plate evaporation. A Unisense oxygen microsensor was used to confirm that O_2_ was present, and wells were well mixed at depth in oxic treatments, including those that contained H_2_S.

### Image processing.

Cells were imaged with an Olympus IX-81 inverted microscope equipped with a long working distance 40× objective (numerical aperture, 0.6; working distance, 2.7 to 4.0 mm) and a 17.28-megapixel DP73 color camera. Images were collected with CellSens dimension (Olympus, Japan) software under constant (manually set) exposure and white balance settings. ImageJ was used to subtract the background and convert the image to an XYZ color profile. A 40- by 40-pixel region of interest representing an area of cytoplasm with a low density of sulfur globules was chosen for quantification of dye change in each cell. The average pixel intensity of this region was then measured in the luminance channel (Y). Reciprocal intensity was calculated as described in reference 49 for quantification of chromogen intensity. The change in intensity relative to that at time zero was reported, and a Student's *t* test was used to determine the likelihood that the imaged intensities were distinct from the mean of the controls by chance alone (Table 1). Copying of the selection region in ImageJ from image to image in the time series for an individual cell ensured that the same area of the cell was measured at each time point. Uniform adjustment of color levels was used for presentation of images in Fig. 1, but this adjustment was performed after image analysis.

### Genome analysis.

To compare our findings to genomic results, *Beggiatoaceae* genomes that were annotated by the IMG pipeline were queried with tools available on IMG/ER (version 4.570 for this study) (50). The queried genomes were *B. leptomitoformis* D-402 (Joint Genome Institute Genomes Online Database [GOLD] Analysis Project Ga0111282), *B. alba* B18LD (Ga0024935), *Ca.* Maribeggiatoa sp. strain Orange Guaymas (Ga0010502), *Beggiatoa* sp. strain PS (Ga0027801), *Beggiatoa* sp. strain SS (Ga0027802), *Ca.* Thiomargarita nelsonii Bud S10 (Ga0097846, Ga0063879), *Ca.* Thiomargarita nelsonii Thio36 (Ga0025452), and *Thioploca ingrica* (Ga0060138). With the exception of the freshwater strains of *Thioploca* (51) and *Beggiatoa*, all genomes are incomplete genomes generated by multiple displacement amplification of individual cells (17, 18, 35, 36).
